# Factors that affect the discontinuation of family planning methods in Myanmar: analysis of the 2015–16 Myanmar Demographic and Health Survey

**DOI:** 10.1186/s40834-020-00126-5

**Published:** 2020-11-10

**Authors:** Khaing Nwe Tin, Thae Maung Maung, Thiri Win

**Affiliations:** 1 Maternal and Reproductive Health Division, Department of Public Health, Ministry of Health and Sports, Naypyitaw, Myanmar; 2 Department of Medical Research, Ministry of Health and Sports, Yangon, Myanmar; 3 Department of Public Health, Ministry of Health and Sports, Naypyitaw, Myanmar

**Keywords:** Contraceptive, Discontinuation, Unmet need, Family planning

## Abstract

**Background:**

Access to family planning contributes up to a 44% reduction in maternal deaths. Since the majority of unplanned pregnancies and abortions occur in women who were either not using contraception or not using it consistently, greater access to contraception and more consistent use of contraception are crucial in the reduction of unplanned pregnancies and abortions. This study aims to determine which types of contraceptives are most often discontinued, the reasons for discontinuation, and the factors that influence contraceptive discontinuation for women in Myanmar.

**Methods:**

This study is a secondary data analysis of calendar data from the 2015–16 Myanmar Demographic Health Survey. The dependent variable is discontinuation of contraception within 12 months among episodes of contraceptive use in the 5 years before the survey among women age 15–49. Multivariable logistic regression was used to identify the predictors of contraceptive discontinuation.

**Results:**

The 12-month discontinuation rate for all contraceptive methods was 39%. The discontinuation rates for short-term methods were remarkably high (43% for pills and 42% for injectables), while the rate for long-term methods was very low (7% for intrauterine devices and 0.2% for implants). Discontinuation while still in need of contraception was high (55%) although 28% of those women switched to other modern methods. Multivariable logistic regression showed the factors associated with contraceptive discontinuation were a woman’s age, location (state/region), wealth, and number of births within the past 5 years.

**Conclusions:**

The high rate of discontinuation while in need is very alarming given goals to reduce the unmet need for family planning in Myanmar. Family planning programs must ensure timely, informed method-switching by women who discontinue contraception, especially among women for whom discontinuation is the highest (age 45–49, middle and richest wealth quintile, regions where high discontinuation and multiparity); increase the availability of long-term contraceptive methods, and improve counseling that ensures clients’ informed and voluntary choice of family planning services.

## Introduction

Improving access to family planning (FP) services is fundamental to achieving the Sustainable Development Goals (SDGs) because it is strongly related with women’s and children’s health, poverty reduction, education, gender equality, and human rights [1]. Access to FP contributes up to a 44% reduction in maternal deaths and a 21% reduction of deaths in children under age 5 [2]. It enhances opportunities for women and girls to attain greater socioeconomic status through education, employment, and empowerment, and accelerates the development of the country by reducing healthcare costs [3].

Myanmar has the second highest maternal mortality ratio (MMR) (178/100,000 live births) among the Association of Southeast Asian Nations (ASEAN^1^) countries [4]. In the 2014 Myanmar Population and Housing Census, the MMR in Myanmar was 282 deaths per 100,000 live births [5]. The pregnancy-related mortality ratio (PRMR) was 227 deaths per 100,000 live births in the 2015–16 Myanmar Demographic and Health Survey (MDHS) [6]. Abortion-related complications were the second leading cause of maternal death [7]. Given these statistics, Myanmar has been working to improve accessibility of modern FP methods to improve maternal and newborn health [8].

Reproductive, maternal, newborn, child, and adolescent health is an explicit public health priority within the Myanmar National Health Plan (2017–2021) [9]. In 2013, Myanmar strongly committed to Family Planning 2020 (FP2020), a global initiative focused on reaching more women with life-saving information and access to contraceptives by the year 2020 and beyond [10].

Myanmar’s FP program began in 1991 as a pilot in a single township. Since 2012, the government has increased its health budget, invested additional resources in the FP program, and increased the accessibility of contraceptives at the community level. Myanmar has worked to increase contraceptive use by married couples and unmarried individuals through informed choice. National surveys showed trends toward an increasing modern contraceptive prevalence rate (CPR) from 38 to 51% and decreasing unmet need for FP from 19 to 16% between 2007 and 2016 [6, 11]. However, these gains are inadequate for achieving the FP2020 targets of greater than 60% CPR and less than 10% unmet need [6, 12]. In addition, there are substantional disparities in unmet need for FP among Myanmar’s states and regions [6].

The majority of unplanned pregnancies and abortions occur among women who were not using contraception or were using it inconsistently, which can lead to poor maternal and child health outcomes, affecting the development of the country [3]. Therefore, greater access to and consistent use of contraception are crucial in the reduction of unplanned pregnancies and abortions.

Contraceptive dynamics, such as discontinuation, switching, and failure, are important markers of how well public health programs are meeting the FP needs of women and couples [13]. Globally, research has focused on determining the reasons for discontinuation of different methods of contraception. Not all discontinuation is necessarily problematic. Some women discontinue a particular method because it is difficult to use or its use is unacceptable to either the woman or her partner, and subsequently switch to another, more suitable method [14]. Other women discontinue because they no longer have a need for contraception. In contrast, women who discontinue contraception despite their desire to limit or delay childbearing represent an extremely important reproductive health problem [15]. Discontinuation for reasons other than “wanted to become pregnant” can lead to unwanted pregnancies, and some of these pregnancies may be terminated by unsafe methods [14]. In a study of 36 low- and middle-income countries [16], 2 % of unintended pregnancies in the last 5 years were due to discontinuation of traditional methods, 18% were due to discontinuation of short-acting modern methods, and 2 % were due to discontinuation of long-term methods. Therefore, reducing premature discontinuation among women who are currently using contraception can be an effective strategy to reduce unmet need and unintended pregnancies [17].

In a study of 25 low and middle income countries, an average 38% of women discontinued contraceptive use within 12 months, 55% within 24 months, and 64% within 36 months. Twelve-month discontinuation rates were highest among condom users (50%) and lowest among intrauterine device (IUD) users (13%), while around 40% of users of the pill, injectables, periodic abstinence, and withdrawal discontinued within 12 months. Discontinuation due to health concerns /side effects, a method related reason, was the most common reason for discontinuation [13]. Studying contraceptive dynamics can identify problems in contraception use and the gaps in service provision [13]. Understanding the reasons for discontinuation and the characteristics of women who discontinue are necessary to evaluate FP strategy and the resource allocation required to ensure the quality of and equitable access to FP services. Although such information is critical for Myanmar’s FP program, there are no prior studies related to contraceptive discontinuation in Myanmar because of a lack of data. The 2015–2016 DHS [6] is the first survey for Myanmar that allows the analysis of contraceptive discontinuation. Thus, this study aims to determine discontinuation rates, the most commonly discontinued methods, reasons for discontinuation, and factors that affect the discontinuation in Myanmar.

## Methodology

This study is a secondary data analysis of the 2015–16 MDHS, a nationally representative survey providing current information on fertility, reproductive health, and FP [6]. The MDHS included information on 12,885 women age 15–49, across 15 States and Regions and urban and rural areas in Myanmar.

Standard recode data files for the MDHS are freely available for access by the public through The DHS Program website, https://www.dhsprogram.com/data/.

Ethics approval for the MDHS was obtained from the Ethics Review Committee of the Department of Medical Research, and Permission for the secondary data analysis presented in this study was obtained from The DHS Program and the Department of Public Health, Ministry of Health and Sports, Myanmar.

This study included information on women, age 15–49, who used contraception during the 5 years before the survey. The data included socio-demographic characteristics and information on FP such as knowledge of contraception, exposure to FP messages and sources of information, and informed decision making on contraceptive use.

### Variables

The dependent variable in the study was twelve-month contraceptive discontinuation which is defined as women age 15–49 who used contraception in 5 years before the survey and discontinued it within 12 months of use.

The independent variables were 5 year age group, marital status (currently married, never married/widowed/divorced/separated), education (highest level attained), residence (urban or rural), state/regions, household wealth quintile, working status (currently working or not), exposure to FP messages (heard about FP on radio, TV, and newspaper or not), contraceptive knowledge (whether respondent has high or low knowledge of any FP method), contraceptive decision making (who made the decision to use contraception: mainly the woman, mainly the man, or both), informed choice of contraceptive use; measured by the method information index, current use of any contraceptive method, the number of children born in the previous 5 years, and the number of living children.

Contraceptive knowledge was calculated as a combined score of the number of contraceptive methods a woman was able to name and her knowledge of the ovulation cycle. There were 12 items in the knowledge score, with high> = 7 and low< 7. The method information index is a proxy indicator for quality of family planning counseling services and informed choice [18]. It ranges from 0 to 3 based on a woman’s “Yes” responses to three variables: 1) the woman was informed about the side effects or problems of the contraceptive method; 2) the woman was informed about what to do if she experienced side effects, and 3) the woman was informed by a health or FP worker of other contraceptive methods that could be used [18].

**Fig. 1:**
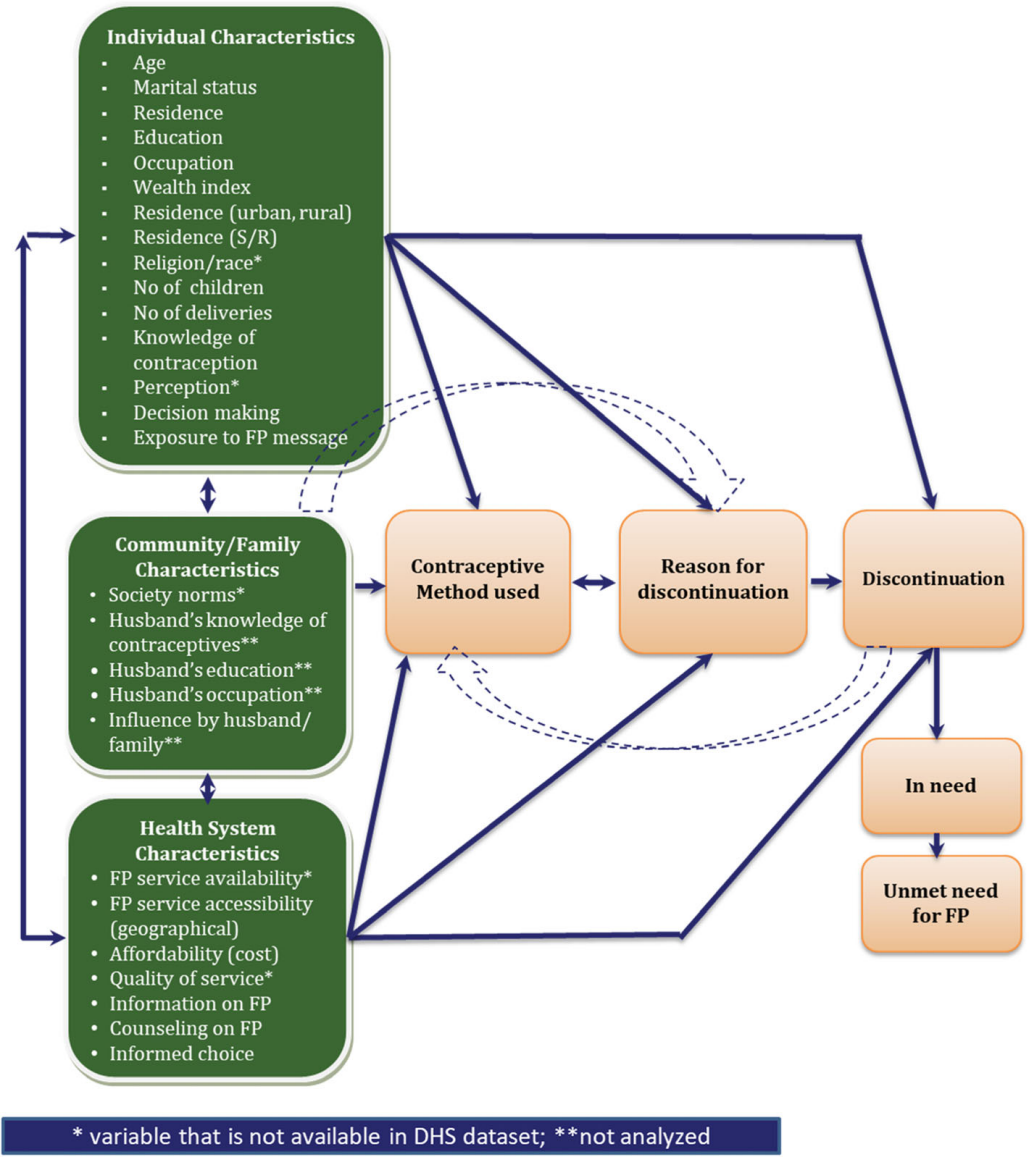
Conceptual framework of the factors that influence contraceptive discontinuation among women age 15–49

The selection of variables was guided by a conceptual framework, shown in Fig. 1, and by the availability of data in the MDHS.

### Conceptual framework

The study team developed the conceptual framework to conceptualize a wide range of factors that could influence contraceptive discontinuation, with reference to studies on discontinuation and contraceptive use [15, 19]. It suggests the factors that influence contraceptive discontinuation among women age 15–49 are individual characteristics, community/family characteristics, health system characteristics, and contraceptive method used.

#### Data management and analysis

To describe contraceptive dynamics, we used DHS calendar [20] data in the woman’s standard recode file, which collected monthly information on contraceptive use and reproductive events retrospectively for the 5 years before the survey. From this data file, the study team created an events file containing 6980 episodes of contraceptive use (the unit of analysis) among women 5431 age 15–49 who used contraception in the 5 years before the survey.

The study used STATA (Version 15 STATA Corp., College Station, TX, USA). All tests were two-sided with a *p*-value of less than 0.05 considered statistically significant. Twelve-month discontinuation rates and reasons for discontinuation are described by frequency and percentages. All possible factors that might influence discontinuation, per the conceptual framework, were included in the bivariate analysis. To identify the factors associated with contraceptive discontinuation, odds ratios (ORs) and their 95% confidence intervals (CIs) were estimated using multivariable logistic regression.

## Results

### Characteristics of contraceptive users

Table 1 shows the socio-demographic characteristics of the sample of women age 15 to 49 who used a contraceptive method within the 5 years before the MDHS. Among contraceptive users (*n* = 5431), 63% were between age 25–39, only 3% were adolescents (age 15–19), and 12% were between age 20–24. Almost all (96%) users were married. Nearly half of the respondents have primary education and only 8% have higher education. One-third of the women were not working, and about 70% resided in rural areas.

**Table 1:** Characteristics of women age 15–49 who used a contraceptive method within 5 years before the 2015–16 MDHS

Characteristics of contraceptive ever users	(*N*=5,431) n	%
**Age group**		
15-19	144	2.5
20-24	665	12.2
25-29	1,058	19.3
30-34	1,158	21.7
35-39	1,146	21.5
40-44	817	14.7
45-49	443	8.1
**Marital status**		
Married	5,252	96.9
Never married/widowed/divorced/separated	179	3.1
**Education level**		
No formal education	638	12.0
Primary	2,481	46.7
Secondary	1,842	32.6
Higher	470	8.7
**Occupation (*n*=5,417)**		
Not working	1,626	29.1
Professional/technical/managerial	289	4.7
Clerical	58	1.1
Sales	996	18.0
Agricultural-self employed	442	8.7
Agricultural-employee	301	5.5
Household and domestic	12	0.2
Services	33	0.7
Skilled manual	312	6.5
Unskilled manual	1,348	25.5
**Wealth index**		
Poorest	1,076	19.8
Poorer	1,100	20.3
Middle	1,069	19.2
Richer	1,126	20.3
Richest	1,060	20.5
**Residence**		
Urban	1,551	28.0
Rural	3,880	72.0
**Region**		
Kachin	278	2.4
Kayah	333	0.5
Kayin	303	2.2
Chin	166	0.4
Sagaing	448	10.8
Tanintharyi	321	2.3
Bago	469	11.1
Magway	362	7.4
Mandalay	393	11.1
Mon	339	3.6
Rakhine	353	5.4
Yangon	454	14.4
Shan	340	10.6
Ayeyarwady	473	15.3
Nay Pyi Taw	399	2.8
**Number of births in last 5 years**		
No birth	2,574	49.8
1	2,355	42.9
2	451	06.5
3	51	00.7
**Number of living children**		
0	449	9.1
1	1,461	28.8
2	1,533	28.9
3	1,012	18.0
4+	976	15.1
**Knowledge on family planning**		
Poor	1,484	27.3
Good	3,947	72.7
**Exposure to FP message/source of information**		
No exposure	3,047	52.5
Exposure from any source	2,384	47.5
**Decision making for contraceptive use**		
Mainly women	1,215	33.0
Mainly husband	75	1.8
Jointly	2,437	64.9
Others	18	0.4
**Informed choice**		
Yes	806	22.6
No	2,873	77.4

About half of the women (53%) did not receive FP messages from any source, while 73% had a good contraceptive knowledge score. The method information index; a proxy indicator for quality of counseling services, was 23%, which means that only two in ten of the contraceptive users had informed choice when they used FP methods. About two-thirds (64%) of the women made the decision to use contraception jointly with their partners, while one-third (33%) made the decision themselves.

### 12 month discontinuation rate

As shown in Table 2, the 12-month discontinuation rate for all methods was 39%. Among the different methods, the discontinuation rate for the lactation amenorrhea method (LAM) was the highest. The discontinuation rates for short-term methods were remarkably high, at 43% for pills and 42% for injectables, while the rate for long-term methods was very low, at 7% for the intrauterine device (IUD) and 0.2% for implants.

**Table 2:** 12-month contraceptive discontinuation rates among episodes of contraceptive use beginning within the 5 years before the 2015–16 MDHS among women age 15–49

Discontinuation by methods	(*N*=6,980) (%)
LAM	84.9
Pill	43.0
Injectable	41.5
All	39.1
Male condom	31.0
Withdrawal	23.0
Periodic abstinence/rhythm	19.5
IUD	7.1
Other	5.4
Implant	0.2

### Reasons for discontinuation

Table 3 shows the reasons for discontinuation. Health concerns/fear of side effects (31%) was the single most common reason for discontinuing the use of a contraceptive method within 12 months.

**Table 3:** Reasons for discontinuation among episodes of contraceptive use beginning within the 5 years before the 2015–16 MDHS among women age 15–49

Reasons for contraceptive discontinuation	(***N***=6,980) (%)
Health concerns/side effects	31.0
Wanted to become pregnant	26.7
Wanted a more effective method	12.5
Other fertility-related reason	12.4
Other method-related	6.3
Became pregnant while using	6.2
Don’t know	4.9

**Fig. 2:**
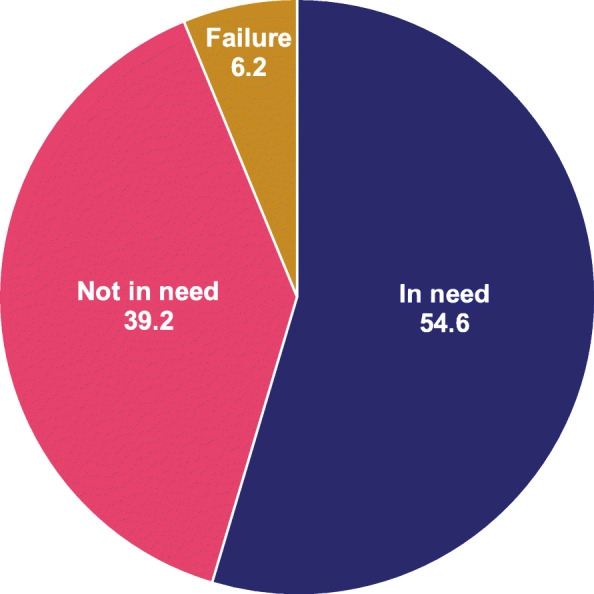
Discontinuation rates by need

These individual reasons can be grouped into *discontinued due to failure*, i.e. became pregnant while using a method; *discontinued due to no need*, such as “wanted to become pregnant” and “other fertility-related” reasons; and *discontinued while in need*, including reasons like “health concerns/side effects” and “wanted a more effective method.” As shown in Fig. 2, almost 55% of the contraceptive users discontinued their contraceptive use within 12 months despite still being in need of contraception.

### Factors influencing the contraceptive discontinuation

In the bivariate analysis, women who were age 45–49, not currently married, technical and clerical employees, in the richest quintile, and use of any contraceptive method other than LARCs were significantly associated with discontinuation within 12 months (Table 4). Residing in selected states and regions was also associated with discontinuation.

**Table 4:** Factors associated with contraception discontinuation among episodes of contraceptive use beginning within the 5 years before the 2015–16 MDHS among women age 15–49

Characteristics	Contraceptive Discontinuation %	Crude OR^**a**^ [95% CI]	aOR^**b**^ [95% CI]
**Age group (*n*=4,982)**			
15-19	23.4	Ref	Ref
20-24	26.33	1.16 [.77-1.76]	1.16 [0.76-1.79]
25-29	24.45	1.05 [.69-1.59]	1.04 [0.68-1.59]
30-34	24.0	1.03 [.67-1.56]	0.97 [0.63-1.49]
35-39	24.1	1.03 [.63-1.70]	0.94 [0.57-1.54]
40-44	31.3	1.48 [.93-2.36]	1.29 [0.82-2.05]
45-49	5.2	3.5* [1.59-7.75]	2.68* [1.31-5.46]
**Marital status (*n*=5,884)**			
Married	25.1	Ref	
Never married/widowed/divorced/separated	95.0	5.47* [3.52-8.49]	
**Education level (*n*=5,884)**			
No formal education	25.5	Ref	
Primary	25.2	0.98 [0.72-1.35]	
Secondary	28.0	1.14 [0.77-1.68]	
Higher	25.8	1.02 [0.64-1.61]	
**Occupation (*n*=4,971)**			
Not working/dependent	22.3	Ref	
Professional/technical/managerial	37.5	2.09* [1.01-4.33]	
Clerical/sales/services	28.6	1.39* [1.02-1.91]	
Agricultural	27.0	1.29 [0.78-2.14]	
Manual worker	26.7	1.27 [1.02-1.58]	
**Wealth index (*n*=4,982)**			
Poorest	23.0	Ref	Ref
Poorer	25.1	1.12 [0.84-1.48]	1.13 [0.84-1.52]
Middle	30.0	1.43 [0.96-2.13]	1.42* [1.02-2.00]
Richer	24.0	1.05 [0.77-1.44]	1.19 [0.86-1.63]
Richest	29.7	1.41* [1.02-1.97]	1.66* [1.18-2.34]
**Residence**			
Urban	26.5	Ref	
Rural	26.1	0.98 [0.74-1.29]	
**Region (*n*=5,884)**			
Yangon	16.4	Ref	Ref
Kachin	21.8	1.41 [0.85-2.36]	1.62* [1.98-2.67]
Kayah	23.5	1.56* [1.05-2.33]	1.79* [1.19-2.68]
Kayin	37.6	3.07* [1.96-4.81]	3.53* [2.29-5.44]
Chin	28.8	2.05* [1.21-3.48]	2.12* [1.25-3.59]
Sagaing	26.8	1.86* [1.23-2.80]	2.06* [1.38-3.10]
Tanintharyi	56.4	6.58* [3.93-11.02]	7.87* [4.61-13.44]
Bago	15.5	0.93 [0.64-1.36]	1.08 [0.74-1.58]
Magway	36.4	2.90* [1.52-5.55]	3.27* [1.81-5.93]
Mandalay	23.7	1.58 [0.98-2.55]	1.80* [1.11-2.93]
Mon	42.3	2.72* [2.35-5.90]	4.03* [2.54-6.41]
Rakhine	34.2	2.64* [1.69-4.12]	3.19* [2.03-5.02]
Shan	32.2	2.42* [1.44-4.06]	2.52* [1.49-4.27]
Ayeyarwaddy	17.5	1.08 [0.72-1.61]	1.35 [0.89-2.02]
Nay Pyi Taw	23.1	1.53* [1.03-2.26]	1.76* [1.17-2.63]
**Number of births in last 5 years (*n*=4,982)**			
No births	30.3	Ref	Ref
1	23.1	0.69* [0.54-0.89]	0.78* [0.62-0.98]
2	28.5	0.92 [0.66-1.29]	1.01 [0.73-1.40]
3	37.6	1.38 [0.85-2.26]	1.66 [0.98-2.82]
**Number of living children (*n*=4,982)**			
0	27.7	Ref	
1	27.2	0.97 [0.73-1.29]	
2	23.5	0.80 [0.57-1.14]	
3	23.0	0.78 [0.55-1.11]	
4+	31.4	1.19 [0.83-1.72]	
**Knowledge on family planning**			
Poor	25.8	Ref	
Good	26.4	1.03 [0.84-1.26]	
**Exposure to FP message/source of information (*n*=4,982)**			
No exposure	26.5	Ref	
Exposure from any source	25.9	0.97 [0.80-1.17]	
**Decision making for contraceptive use (*n*=3,494)**			
Mainly women	18.0	Ref	
Mainly husband	12.1	0.62 [0.19-2.01]	
Jointly	14.6	0.78 [0.54-1.11]	
**Informed choice**			
No	15.6	Ref	
Yes	15.9	1.02 [0.74-1.40]	
**By methods(*n*=1,458)**			
LARC	2.0	Ref	
Pills	31.0	22.40* [5.24-95.4]	
Injection	27.3	18.74* [4.43-79.18]	
Other modern methods including condom	14.1	8.17* [1.70-39.13]	
Traditional method	12.6	7.17* [1.30-39.47]	

*p<0.05

^a^crude odds ratios from binomial logistic regression

^b^adjusted odds ratio from multivariate logistic regression

After controlling for the other factors with multivariable logistic regression, the factors associated with contraceptive discontinuation were the women’s age group, location (state/region), wealth quintile, and number of births within 5 years. Women aged 45–49 (OR = 2.68; 95%CI: 1.31 to 5.46) and those from the middle (OR = 1.42; 95%CI: 1.02 to 2.00) and richest quintile group (OR = 1.66; 95%CI: 1.18 to 2.34) were more likely to discontinue contraceptive use. Women with one child (OR = 0.78; 95%CI: 0.62 to 0.98) were 22% less likely to discontinue the contraceptive use. With the exception of Bago and Ayeyarwaddy, residing in any other state or region was found to be significantly and positively associated with discontinuation compared with Yangon. Residents of Tanintharyi had nearly 8 times the odds of discontinuation (OR = 7.8; 95%CI: 4.61 to 13.44) as residents of Yangon (Table 4).

## Discussion

This study was a secondary analysis of the first nationally representative 2015–16 MDHS of 12,885 women age 15–49 from both urban and rural areas in Myanmar. The findings have a number of policy and practical implications for the national FP program because the MDHS was nationally representative. Among the 5431 women used contraception in the preceding 5 years, nearly three-fourths had a good knowledge score, which is the combined score of a woman’s ability to name contraceptive methods and to know the fertility period. In the MDHS [6], 97% of all women had heard of a contraceptive method, which was consistent with the 2007 Fertility and Reproductive Health Survey, in which knowledge of FP was 95% [11]. However, in a small Myanmar study, only 20% of married women had a knowledge score that reflected detailed information of contraceptive usage such as eligibility, benefits, side effects, and warning signs of different methods (Tin KN, Mon MM: Task sharing to Auxiliary Midwives for Family Planning Services in selected hard-to-reach areas in Myanmar, unpublished). The MDHS found that about half of women who used contraception in the 5 years before the survey did not receive FP messages from any source [6]. This highlights a high contraceptive knowledge score is not necessarily equated with having comprehensive FP information and the ability to make informed choices regarding contraception. Therefore, it is necessary to strengthen the provision of comprehensive FP information so that women can be more knowledgeable about method choices, and more likely to choose a method satisfactory to them that they can use for a sustained period of time [21].

In this study, the 12-month contraceptive discontinuation rate among women age 15–49 who started an episode of contraceptive use within the 5 years before the MDHS was 39% for all methods., This rate was consistent with the average 12-month discontinuation rate (38%) in 25 other low and middle income countries [13]. The discontinuation rate in this study is similar to the rates of 38% in Bangladesh, 34% in Senegal, 31% in Tanzania and 27% in Ethiopia [22–25], indicating that Myanmar’s contraceptive discontinuation magnitude is similarly high as other developing countries.

In addition, discontinuation rates varied by method, with discontinuation of short-term methods being higher than long-term methods. This study found a 12-month discontinuation rate of about 40% for pills or injectables versus 7% for the IUD [6]. These results are similar to the method-specific discontinuation rates found in the 25-country study (40% for pills and injectables versus 13% for IUD) [13]. The MDHS results were also consistent with a study from Senegal, in which discontinuation was lower among long-term methods (6 and 18% for implants and IUD, respectively) than for pills (38%) and injectables (33%) [23]. Likewise in an Ethiopian study, the highest discontinuation rates were found in pill users (38%) while IUD users had the lowest discontinuation rates (14%) [25]. Short-term method users may discontinue at higher rates because they can stop using their method without the assistance of health providers. Additionally, in Myanmar, short-term methods are readily available at both public and private health facilities, pharmacies, drug shops, and retailers in some rural communities. In hard-to-reach areas, untrained community health volunteers provide injections of Depo-Provera, though without providing any comprehensive user information [26]. In the 2015–16 MDHS, the source for pills was the private sector (47%) and other sources (39%), while the source for injectables was mainly the public sector (74%) [6].

In the MDHS, the method information index; an indicator of quality counseling, for all methods was 23%. For short-term methods, the index was quite low (13% for pills and 26% for injectables), compared with long-term methods (64% for implants and 53% for IUD) [6]. This suggests that short-term methods may be given without full counseling and information in both the public and private sectors. This also highlights the critical importance of proper restrictions on selling injections to unskilled individuals, comprehensive information sharing with clients by drug shops and pharmacies, awareness training on FP for drug sellers, as well as quality counseling and adoption of informed choice principles by all healthcare providers.

In this study, the primary reasons for discontinuation were health concerns or fear of side effects. This was similar to the evidence from other studies, which similarly found health concerns/ side effects to be the most common reason and that counseling was inadequate [14, 16]. In a study in Bangladesh, it was also found that the experience of side effects was the main reason for discontinuation [27]. Although the reasons can differ between users of short and long acting methods, this study found that health concerns/side effects was the most common reason for both types of methods. One limitation of this study which uses DHS data, is that we cannot distinguish between discontinuation because women actually experienced health problems and discontinuation due to their fear of side effects. This should be explored with qualitative interviews.

In this study, discontinuation due to method failure was low (6%), which was similar to the findings of a study in Pakistan (8%) [28] but lower than failure rates found in another multi-country study [16]. Because perfect use failure rates are estimated to be less than 1% for all methods [14, 21], the magnitude of method failure in Myanmar might be a result of inadequate counseling or incorrect information on correct and effective use [29].

Discontinuation while still in need of contraception was high in this study (55%). However, 28% of those women reported switching to other modern methods. High discontinuation while still need of contraception is alarming in light of the country’s efforts to reduce unmet need for family planning and to prevent unwanted pregnancies. Family planning programs must ensure timely, accurate information on alternative methods to those women who discontinue contraception in order to prevent unintended pregnancies and unsafe abortion [14].

According to the MDHS, unmet need for FP in Myanmar was 16%, comprised of 11% who had an unmet need for limiting and 5% who had an unmet need for spacing. There is considerably high use (68.4%) of short-term methods (pills or injectables) in Myanmar, given the large proportion of women who want to limit births [6]. This reliance on short-term methods may be unsustainable and contribute to discontinuation, indicating a need to improve service availability, quality and counseling to help women make an informed choice.

When identifying the factors that influence discontinuation, women age 45–49, from the middle and richest wealth quintile, location (all states and regions except Bago and Ayeyarwaddy) were positively associated and having one birth in the past 5 years was negatively associated with discontinuation after controlling for other possible confounding factors. It is worth exploring the reasons for the high discontinuation rates found in the Taninthari region. In a similar analysis of the 2013 Nigeria DHS, the predictors for discontinuation were women’s age, residence, education, number of children under age 5, marital duration, women’s occupation, men’s occupation, and wealth [19]. Family size and number of children were associated with discontinuation in a Pakistan study [28].

Among the factors associated with contraceptive discontinuation in bivariate regression, the method used was the most important factor; it could not be included in multivariate analysis due to the small sample size of IUD users. In this study, women who used short-term methods and traditional methods were more likely to discontinue than long-term methods users, which is consistent with studies in other countries [13, 14]. Thus, information and counseling should be more focused to short-term method users to prevent discontinuation while in need. In addition, there might be other potential family/community and health service factors that might influence contraceptive discontinuation which could not be identified in this study. Further qualitative research could explore these gaps.

The method information index was high for long-term method users but quite low for short-term method users. It is possible that long-term contraceptives are provided by trained medical personnel who provide professional counseling. Although there was not a significant association between discontinuation and the method information index, after controlling for the method used, FP programs should promote the availability of long-term methods through effective counseling to make informed choice that assure sustained contraception. Long-term methods are cost-effective methods as compared with short-term contraceptives, and align with Myanmar FP2020 commitments to increase access to and consistent use of contraception in Myanmar [12, 30].

### Strengths and limitations

This study utilized data from the 2015–16 MDHS, the first nationwide representative survey conducted in Myanmar. The study used DHS-standardized data collection tools, data management strategies, and data analysis techniques, which generate nationally representative data that can be compared with data from other countries where DHS were conducted. The study had some limitations. Husband/family/community factors and some health system factors (service availability, accessibility, and quality of services) were not assessed in the study because this information was not available in the MDHS. Since this study was based on cross-sectional data, possible causal associations with some factors is limited, which limits our understanding of individuals’ and couples’ experiences that contribute to contraceptive discontinuation.

## Conclusion

The 12-month discontinuation rate for all methods was high, primarily due to discontinuation of short-term methods. Discontinuation while in need was also considerably high, with health concerns/fear of side effects cited as the most common reason. While women’s age group, location (state/region), wealth, and number of births within 5 years were significantly associated with discontinuation, the method information index was not statistically significant in this study. Timely and accurate information on method alternatives should be promoted to reduce contraceptive discontinuation while women are in need and promote informed choice, especially among older (age 45–49) women and women with multiple births, and in regions which have high discontinuation rates. The Myanmar FP program must also improve the availability of contraception including long-term methods to ensure clients can choose their preferred method from a range of choices through effective counseling and voluntary FP services.

## Data Availability

The DHS data is freely available for access by the public through The DHS Program website, https://www.dhsprogram.com/data/available-datasets.cfm .

## Abbreviations

ASEAN: Association of Southeast Asian Nations
CPR: Contraceptive prevalence rate
CI: Confidence intervals
DHS: Demographic and Health Survey
FP: Family planning
FP2020: Family Planning 2020
IUD: Intrauterine device
LAM: Lactation amenorrhea method
MDHS: Myanmar Demographic Health Survey
MII: Method information index
MMR: Maternal mortality ratio
OR: Odds ratio
PRMR: Pregnancy related mortality ratio
SDGs: Sustainable Development Goals

## Footnotes

1 Members of ASEAN countries are Brunei, Cambodia, Indonesia, Laos, Malaysia, Myanmar, Philippines, Singapore, Thailand and Vietnam.
